# Antibodies reacting with Simian Virus 40 capsid protein mimotopes in serum samples from patients affected by uveal melanoma

**DOI:** 10.1186/1756-8722-7-38

**Published:** 2014-04-29

**Authors:** Ilaria Bononi, Paolo Perri, Alice Begnardi, Alessandra Martini, Elisa Mazzoni, Silvia Bosi, Silvia Pietrobon, Adolfo Sebastiani, Mauro Tognon, Fernanda Martini

**Affiliations:** 1 Department of Morphology, Surgery and Experimental Medicine, School of Medicine, University of Ferrara, Via Fossato di Mortara 64/b, Ferrara 44121, Italy; 2 Department of Biomedical Sciences and Surgical Specialities, School of Medicine, University of Ferrara, Via Luigi Borsari 46, Ferrara 44121, Italy

**Keywords:** Uveal melanoma, Simian Virus 40, ELISA, Antibody, Prevalence

## Abstract

The uveal melanoma (UM) is the most common human intraocular tumour. Simian Virus 40 (SV-40) is a small DNA tumor virus detected in some malignancies, including the cutaneous melanoma. In this study an indirect ELISA using synthetic peptides that mimic SV-40 antigens, was employed to detect antibodies against SV-40 in serum samples from UM patients. Our report indicates a significant higher prevalence of antibodies against SV-40 capsid protein antigens in serum samples from UM patients compared to controls. Our data suggest an association between UM and SV-40, indicating that patients affected by uveal melanoma tested SV-40-positive could be treated by innovative therapies.

## Findings

The uveal melanoma (UM) is the most common primary intraocular tumor. Many studies reported genetic alterations in UM [1], but the causes are poorly understood. BAP1, a gene encoding a deubiquitinant enzyme, is mutated in several UM cases and in the malignant pleural mesothelioma (MPM) [2], a human tumour found be associated with the Simian Virus 40 (SV-40) infection [3,4]. SV-40 oncogenic potential has been demonstrated in experimental animals [3,4], while its mutagenic activities have been detected in different animal and human cell types [3,4]. In human tumours, SV-40 was identified for the first time in a patient affected by a cutaneous melanoma [5], that shares the onset model with UM. Altogether these data were the background that prompted us to investigate the association between UM and SV-40 by analysing the prevalence of SV-40 antibodies in serum samples from UM affected patients. This study was carried out, as reported before, by an indirect Enzyme-Linked Immunosorbent Assay (ELISA) with SV-40 specific synthetic peptides derived from its viral proteins, without cross-reactivity with the closely related BKV and JCV which are obiquitous polyomaviruses in humans [6].

In this investigation, serum samples from UM affected patients (n = 48) and healthy subjects with ocular nevi (HSON; n = 71) and without ocular nevi (HS; n = 168), with the same median age (66 yrs), were analysed for presence of SV-40 antibodies. All patients and subjects were vaccinated against the poliomyelitis. The immunologic study was carried out by indirect ELISAs employing two specific mimotopes from SV-40 viral capsid proteins 1 and 2–3, named B and C peptides, respectively [6]. In our experiments, serum samples were considered SV-40 VP-positive upon reacting to both peptides B and C. Informed written consent was obtained from the patients and subjects. The study was approved by the County Ethical Committee, Ferrara, Italy.

**Figure 1:**
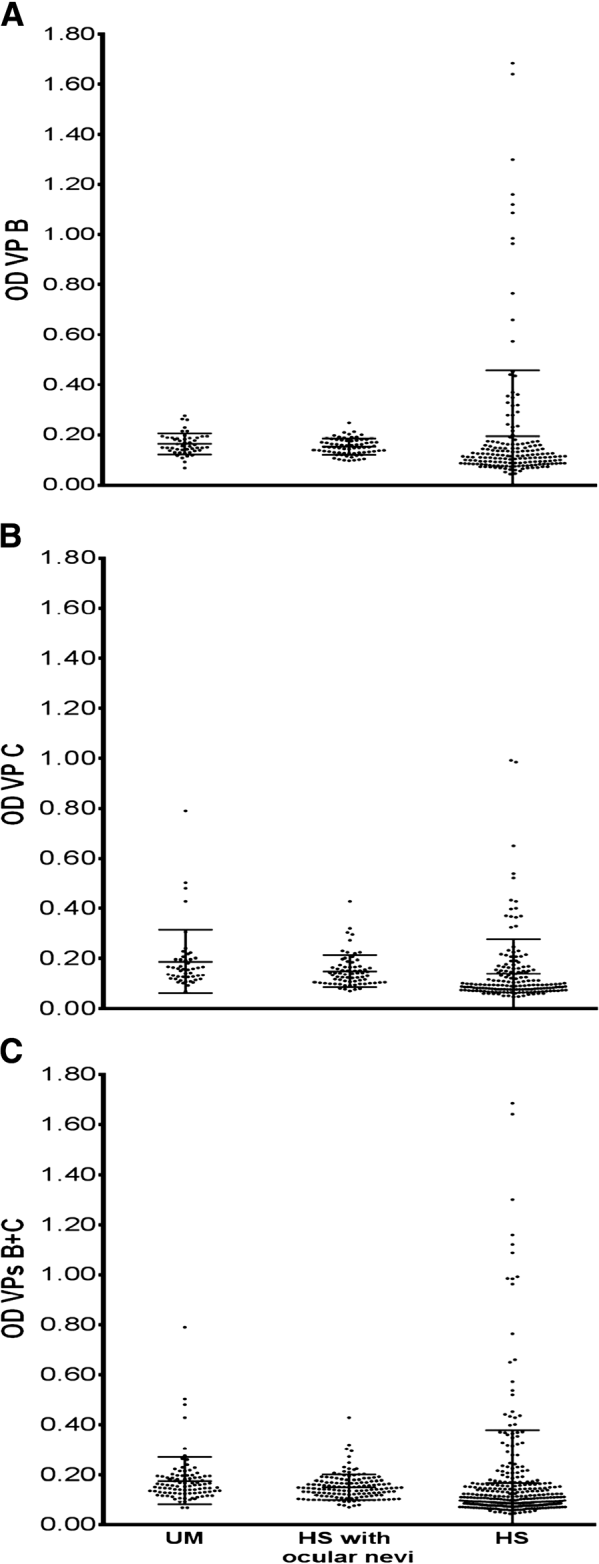
**Serologic profile.** Serologic profile of serum antibody reactivity to SV-40 mimotopes VP1 B **(panel A)** and VP2/3 C **(panel B)** and VPs B + C **(panel C)**. Immunologic data are from patients affected by UM and from healthy individuals (HS) with and without ocular nevi. Data are OD values at 405 nm of serum samples diluted 1:20, detected in indirect ELISA. In scatter dot plotting, each plot represents the dispersion of OD values to a mean level indicated by the line inside the scatter with standard error mean (SEM) for each group of subjects analyzed (Mean OD ± SEM).

The overall prevalence, by combining SV-40-positive sera for both VP1 B and VP2/3 C peptides, in UM patients was 33%, higher than that detected in HSON or HS, 17% and 15% respectively. The difference between UM patients and HSON or HS is statistically significant (p = 0.038 and p = 0.004, respectively). Serologic profiles of serum antibody reactivity to SV40 mimotopes are reported in Figure 1. The difference of OD=optical density mean value of sera from UM and two control groups, is not statistically significant (p > 0.05).

Our immunologic data indicate that a subset (1/3) of UM is associated with SV-40, a small DNA tumour virus detected as a contaminant in early anti-polio vaccines [3,4,7]. At present, SV-40 infection seems to spread in humans by different ways, including the urine and the faecal-oral route [3,4,7].

UM onset, like other human cancers, is due to specific gene mutations. Since SV-40 is oncogenic, clastogenic, mutagenic and a transforming viral agent [3,7], may be a risk factor, together with other oncogenic agents such as the U.V. irradiation, in the UM onset/progression [8]. One may postulate that after infecting the host, SV-40 may exert its tumourigenic potential when the immune system is impaired. The high prevalence of SV-40 antibodies in sera from UM affected patients is not proof of cause/effect in inducing human tumours by SV-40. SV-40 DNA and expression of its oncogene, the large T antigen, should be analysed in UM specimens to confirm and extend the potential role of this oncogenic virus in UM onset/progression. We should also consider, as an alternative explanation, that another not yet discovered human Polyomavirus closely related to SV-40 may be responsible of our immunologic data. Our results from the laboratory bench could be transferred to the clinical application employing specific innovative therapies for SV40-positive UM patients.

## Competing interests

The author declare that they have no competing interests.

## Authors’ contributions

TM, SA and MF designed the study and secured funding; MF, TM, PP and SA given final approval of the version to be published; PP, MA and BS collected samples; PP and SA performed the clinical diagnosis; BI, BS and BA conducted the experimental work; BI, BA, MF and TM analysed the data and wrote the manuscript; ME and PS gave support in analysis of data and statistics; MF and TM made the final critical revision. All authors read and approved the final manuscript.
